# Nonlinear Material Model for Quasi-Unidirectional Woven Composite Accounting for Viscoelastic, Viscous Deformation, and Stiffness Reduction

**DOI:** 10.3390/polym10080903

**Published:** 2018-08-11

**Authors:** Zhanyu Zhai, Bingyan Jiang, Dietmar Drummer

**Affiliations:** 1 Institute of Polymer Technology, University Erlangen-Nuremberg, 91054 Erlangen, Germany; dummer@lkt.uni-erlangen.de; 2 State Key Laboratory of High Performance and Complex Manufacturing, Central South University, Changsha 410083, China; jby@csu.edu.cn

**Keywords:** viscoelastic strain, viscous strain, composites

## Abstract

To clarify the individual contribution of viscoelastic and viscous deformation to the global nonlinear response of composites, multilevel cyclic loading-unloading recovery tensile tests were carried out. The experimental results show that there is a linear relationship between the viscous strain and viscoelastic strain of composites, regardless of the off-axis angle or loading stress level. On the basis of experimental results, a coupled damage-plasticity constitutive model was proposed. In this model, the plasticity theory was adopted to assess the evolution of viscous strains. The viscoelastic strain was represented as a linear function of viscous strains. Moreover, the Weibull function of the effective stress was introduced to evaluate the damage variables in terms of stiffness reduction. The tensile stress-strain curves, predicted by the proposed model, showed a good agreement with experimental results.

## 1. Introduction

Except when loaded in the fiber direction, fiber reinforced polymer composites (FRPCs) show nonlinear behavior [1]. During the last decades, many attempts have been made to characterize this nonlinearity. The general aim of most of these works is to develop a mathematical formulation, which can describe the nonlinear stress-strain curves of FRPCs under off-axis loading.A number of different models have been proposed by various authors. They can be generally distinguished between microand macromechanical approaches. In terms of the microscopic approach, some details, like the microstructure and the properties of the matrix-fiber interface, are taken into consideration for micromechanical models [2,3,4,5]. From the viewpoint of material development, the micromechanical models can provide a better insight into the contributions of each constituent to the macromechanical properties. However, many researchers have shown that modeling the interface and measuring the properties of the constituents remains challenging work. Moreover, micromechanical models usually require high computational resources.

In the macroscopic scale, modeling is on the ply level. Some macromechanical models were built based on the nonlinear elasticity theory [6,7], progressive damage-elasticity theory [8,9], elasticity-plasticity theory [10,11], and the coupled damage-plasticity theory [12,13,14,15]. Among these models, the one-parameter elasticity-plasticity model [11], working with a plastic potential function, is mostly adopted to characterize the nonlinearity in composites due to its simplicity. However, it overestimates the plastic deformation of FRPCs since the model fails to identify the nonlinear elastic deformation that is caused by damage. In addition, the coupled damage-plasticity model proposed by Ladeveze and Dantec [15] is also widely employed and developed to describe the nonlinear response of FRPCs, since the identification of model parameters is easier. In the coupled damage-plasticity model, the nonlinearity in composites was assumed to be caused by the plastic deformation and stiffness reduction. To be specific, the damage variable was defined as the reduction in material stiffness. The plasticity model was introduced to evaluate the plastic strain. It should be noted that the plastic strain in the above macromechanical models was assumed to be viscous deformation (permanent deformation). However, as described by Marklund et al. [16], there are three distinct phenomena for FRPCs that are caused by the evolution of damage: (1) Micro damage evolution would lead to the reduction in stiffness; (2) sub-micro damage may result in macro viscous behavior; and (3) nonlinear viscoelastic behavior may be attributed to both constituents in composites. Correspondingly, the plastic deformation in the aforementioned macromechanical models should be composed of two parts, i.e., time-dependent reversible plastic strain (viscoelastic strain) and irreversible plastic strain (viscous strain). In the present work, the nonlinear behavior in a quasi-unidirectional (UD) woven composite was described based on the coupled damage-plasticity theory. Especially, the plastic deformation consisting of viscoelastic and viscous deformation in a quasi-unidirectional (UD) woven composite, under off-axis tensile loading, was first observed by performing multilevel cyclic loading-unloading recovery tests. Then, based on the coupled damage-plasticity theory, a new supposed nonlinear constitutive model was built for a quasi-UD woven composite, which can identify the respective parts of elastic, viscoelastic, and viscous deformation in its global nonlinear response. In addition, the evolution of viscoelastic and viscous strains, with loading stress, were determined. Finally, the validity of the developed model was evaluated on the basis of the experimental results that were obtained from monotonic tensile tests.

## 2. Experiments 

### 2.1. Materials and Test Methods

**Figure 1:**
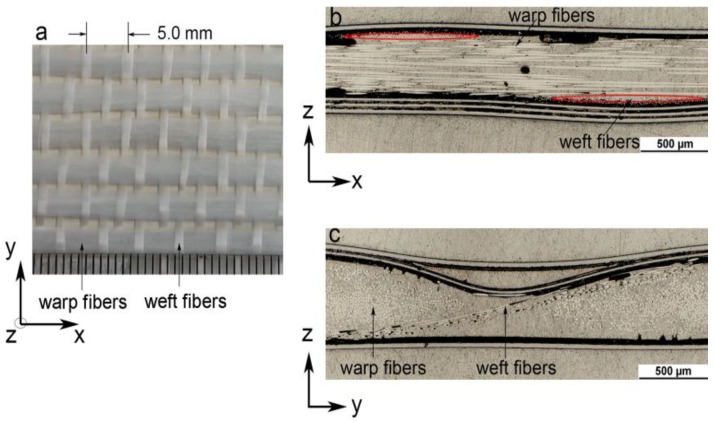
(**a**) Diagram of a quasi-UD woven fabric, observed on the top sight (z direction), (**b**) characteristics of the woven structure in the quasi-UD fabric in the weft direction, and (**c**) characteristics of the woven structure in the quasi-UD fabric in the warp direction.

The material system that was used in this study was a quasi-UD E-glass woven fabric reinforced polypropylene composite (quasi-UD E-glass/polypropylene composite). A quasi-unidirectional woven fabric (GW324-315L, P-D Glasseiden GmbH, Oschatz, Germany) with an areal weight of 315 g/m^2^ was used for reinforcement. It contains 92% vol. of warp fibers and 8% vol of weft fibers, as shown in Figure 1. The polypropylene copolymer (Moplen EP500V) in granule form was provided by the LyondellBasell company (Frankfurt, Germany). The flat film of polypropylene with a 0.21 thickness was first extruded.

A quasi-UD E-glass/polypropylene composite, which is composed of three plies of polypropylene film and two plies of quasi-UD fabric alternatively, was manufactured through a film-stacking technique. The thickness and fiber volume fractions are 0.95 ± 0.07 mm and 33.3% ± 0.5%, respectively. 

Three kinds of testing samples were prepared at off-axis angles, θ (between the loading direction and warp fiber direction), of 10°, 20°, and 90°, which were cut out of a quasi-UD E-glass/polypropylene composite using a water-cooled sawing machine. The shapes and dimensions of the samples are based on the testing standard of ISO 527-4. End tabs were bonded to both the top and bottom sample surfaces in the gripping areas. To be specific, to reduce the end-constrain effect caused by rigid clamps, the oblique end-tabs were adopted for 10° and 20° off-axis samples. The detailed information has been described in our previous work [17].

Multilevel cyclic loading-unloading recovery tests were performed on the samples. Four peak stresses, i.e., around 50%, 60%, 70%, and 80% of its axial tensile strength, were chosen. The axial tensile strength of 10°, 20°, and 90° off-axis samples are available in our previous work [17]. In each loading-unloading recovery cycle, a sample was firstly loaded to the peak stress with the loading speed of 1 mm/min, then unloaded to the point of zero stress with the loading speed of 200 mm/min, and finally held in the zero state for 1800 s for the recovery of viscoelastic strain.

### 2.2. Experimental Results

**Figure 2:**
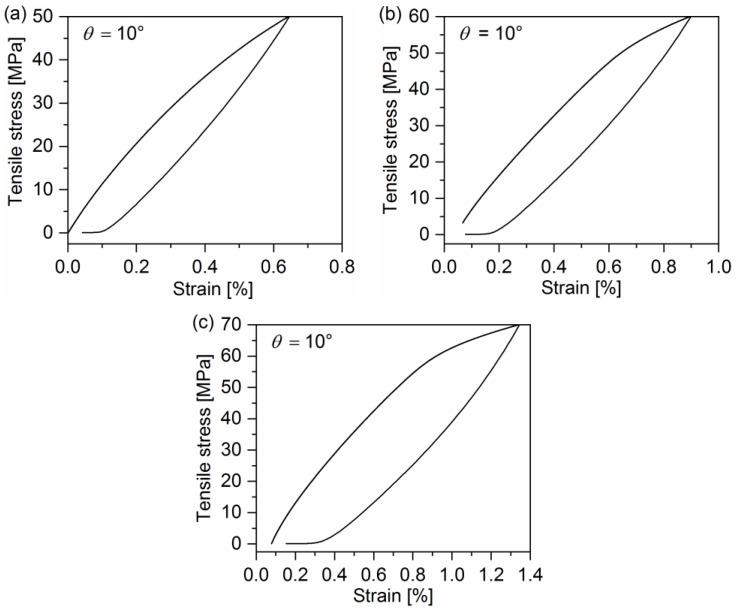
Representative curves from multilevel cyclic loading-unloading recovery tests for the 10° off-axis sample: (**a**) Peak stress: 50 MPa, (**b**) peak stress: 60 MPa, and (**c**) peak stress: 70 MPa.

**Figure 3:**
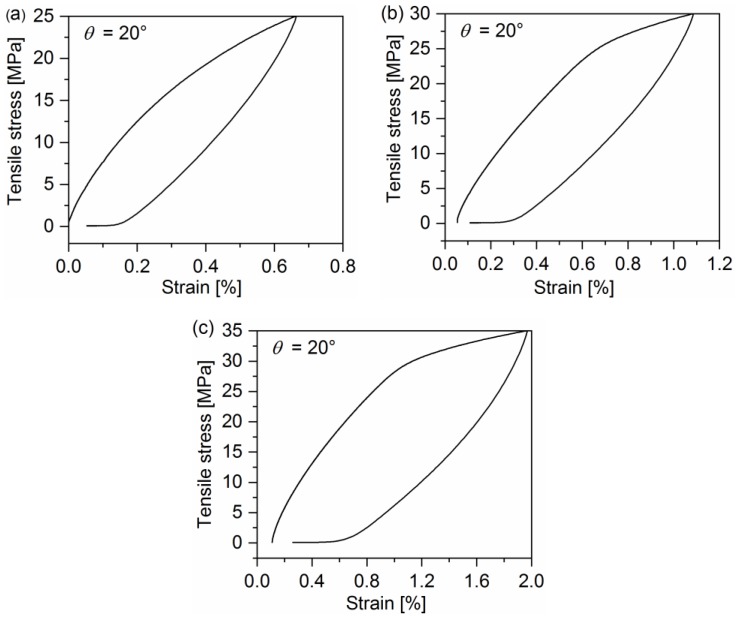
Representative curves from multilevel cyclic loading-unloading recovery tests for the 20° off-axis sample: (**a**) Peak stress: 25 MPa, (**b**) peak stress: 30 MPa, and (**c**) peak stress: 35 MPa.

**Figure 4:**
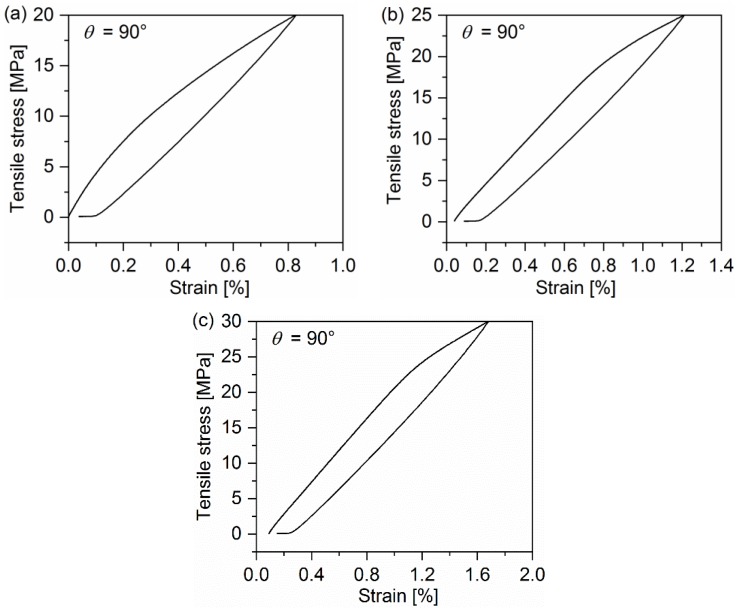
Representative curves from multilevel cyclic loading-unloading recovery tests for the 90° off-axis sample: (**a**) Peak stress: 20 MPa, (**b**) peak stress: 25 MPa and (**c**) peak stress: 30 MPa.

Figure 2, Figure 3 and Figure 4 display the representative curves from multilevel cyclic loading-unloading recovery tests for 10°, 20°, and 90° off-axis samples. Several important features can be observed from cyclic loading-unloading recovery behavior. First of all, the hysteresis loop of off-axis samples is apparent. The second feature is the strain of off-axis samples at the start point of each cycle, which is not equal to zero except for the first cycle. Apparently, the plastic strain after the unloading is not fully recovered in each cycle. It indicates that certain viscous deformations occurred in the quasi-UD E-glass/polypropylene composite under off-axis loading. In addition, the stiffness reduction in off-axis samples after every cycle can be observed.

**Figure 5:**
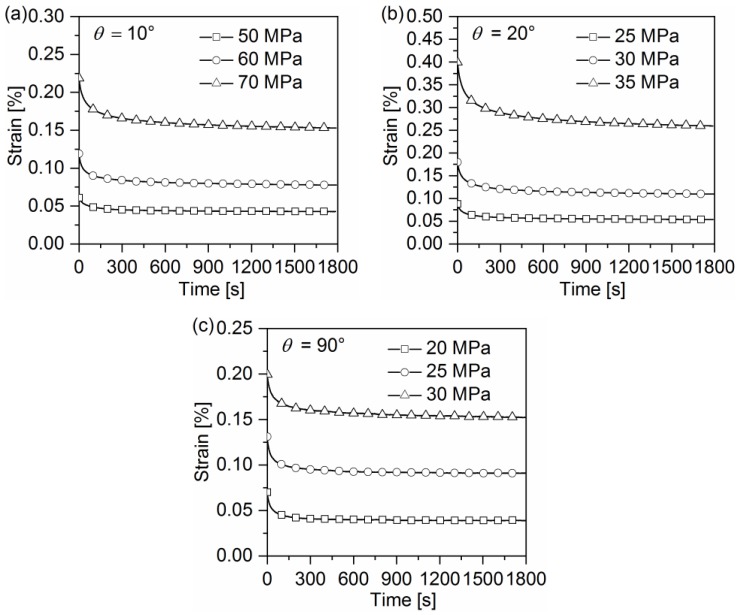
Strain-time curves of the quasi-UD E-glass/polypropylene composite for the strain recovery at zero stress point and with various peak stresses: (**a**) 10° off-axis sample, (**b**) 20° off-axis sample, and (**c**) 90° off-axis sample.

Another important feature can be found in Figure 5a–c, which presents the strain-time curves of off-axis samples for the strain recovery at zero stress point and with various peak stresses. As can be seen, when an off-axis sample is held at the zero stress holding after unloading, the plastic strain recovers obviously. However, the recovery rate decreases gradually with the increasing holding time. A saturation of viscoelastic recovery is almost reached after holding for 600 s. Then, the remaining strain stays almost unchanged with the increasing holding time at zero stress, which is assumed to be the irrecoverable viscous strain that is generated after each cycle. Moreover, it can be found that the irrecoverable viscous strain increases with the increasing peak stress. That can be understood since high stress levels would induce the yielding of material.The viscous and viscoelastic deformation of quasi-UD composites can be observed to be dependent onthe off-axis angle. The viscous and viscoelastic deformation of the 20° off-axis sample is apparently larger than that of the 10° and 90° off-axis samples. This may be due to the different fracture mechanisms in off-axis samples, since the viscous and viscoelastic deformation of composites are caused by the matrix cracks and the fiber-matrix interface debonding.

**Figure 6:**
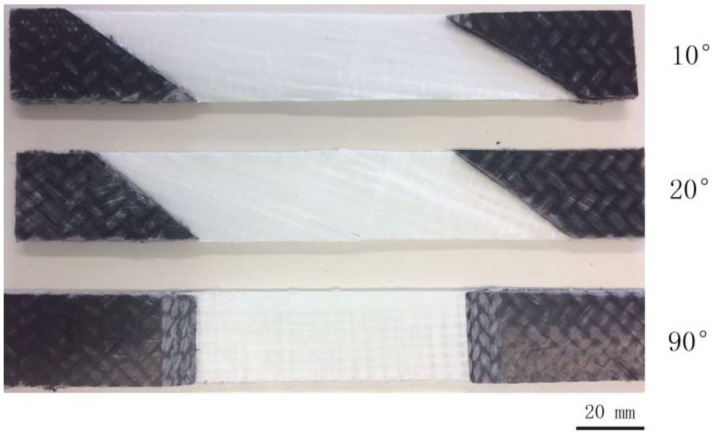
Macroscopic photographs of off-axis samples after the cyclic loading-unloading recovery test.

**Figure 7:**
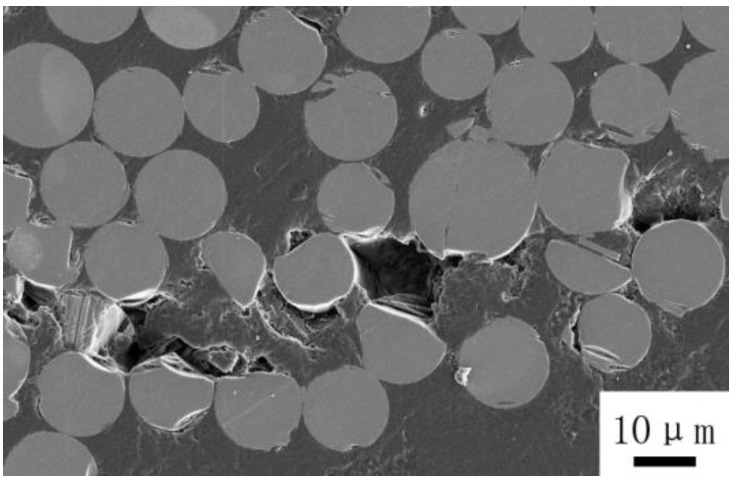
SEM image forthe 10° off-axis sample after thecyclic loading-unloading recovery test.

Macroscopic photographs of off-axis samples after cyclic loading-unloading recovery tests are given in Figure 6. As can be seen, no obvious damage occurred during the test, except for the cracks that are initiated on the edge of the samples. Figure 7 is the scanning electron microscopy (SEM) image of the cross-section of the 10° off-axis sample shown in Figure 6. It can be found that the matrix cracks and interface debonding could not close fully after the recovery process. Correspondingly, irrecoverable viscous strains are observed in Figure 5.

## 3. Nonlinear Material Modeling

**Figure 8:**
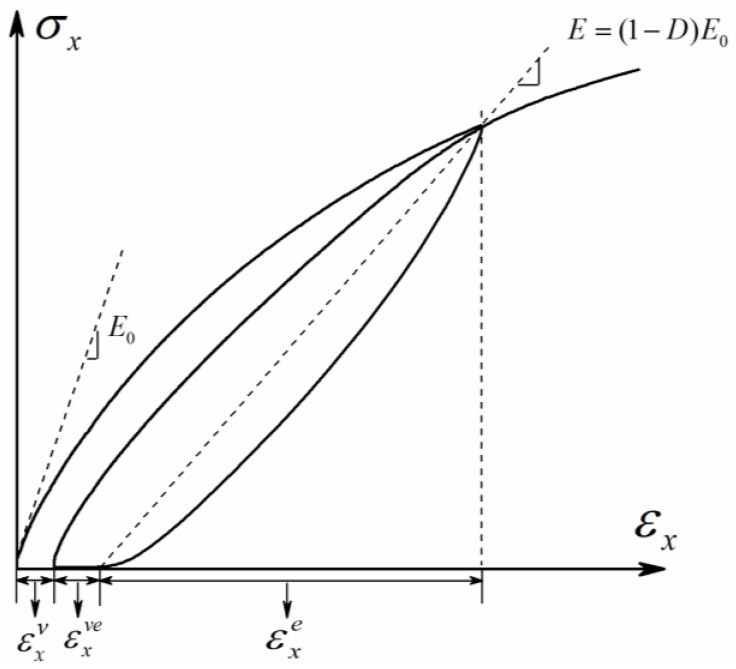
Determination of the damage variable, viscoelastic and viscous strains through cyclic loading-unloading recovery tests.

The experimental results shown in Section 2.2 indicate that the nonlinearity in the off-axis stress-strain curves of quasi-UD composites is caused by the stiffness reduction and the combination of viscous and viscoelastic deformation. Thus, quasi-UD compositesare assumed to be the elastic-damage-plastic material. The incremental axial strain can be composed of three parts:

(1) $$d\varepsilon_{x}=d\varepsilon_{x}^{e}+d\varepsilon_{x}^{v}+d\varepsilon_{x}^{ve}$$

where $\varepsilon_{x}$ is the macro axial strain in the loading direction x; $\varepsilon_{x}^{e}$ is the elastic strain; $\varepsilon_{x}^{v}$ is the viscous strain; and $\varepsilon_{x}^{ve}$ is the viscoelastic strain. Viscous strain and viscoelastic strain can be determined from cyclic loading-unloading recovery tests, as shown in Figure 8.

Taking the same approach with the continuum damage mechanics (CDM) proposed by Ladeveze [15], the elastic damage variable is introduced to evaluatethe reduction in material stiffness. Correspondingly, Equation (1) can be rewritten as:

(2) $$d\varepsilon_{x}=\frac{d\sigma_{x}}{\left(1-D\right)E_{0}}+d\varepsilon_{x}^{v}+d\varepsilon_{x}^{ve}$$

where $\sigma_{x}$ is the macro axial stress; $E_{0}$ is the elastic modulus of undamaged material; and D is the elastic damage variable.

### 3.1. Viscous Strain

**Figure 9:**
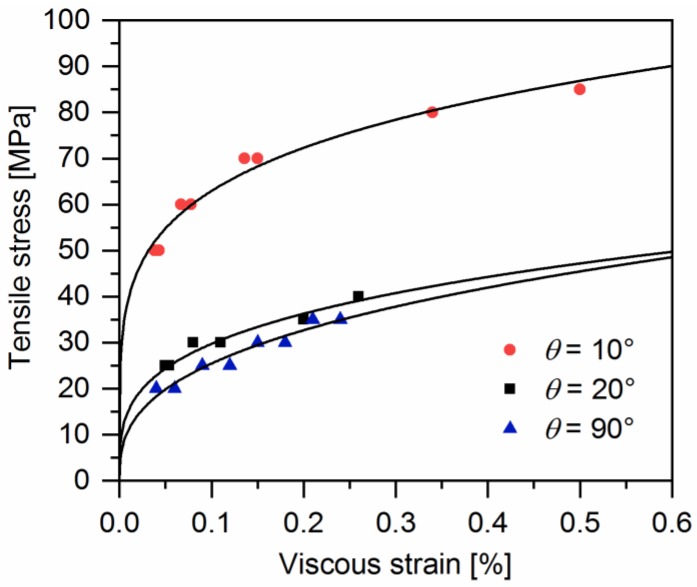
Experimental data of the axial stress-viscous strain of a quasi-UD E-glass/polypropylene composite subjected to off-axis tensile loading.

Axial viscous strains of off-axis samples were measured after each loading-unloading recovery procedure. Figure 9 illustrates the axial viscous strain of samples at the peak loading stress for every cycle. As can be seen, the evolution of viscous strain with loading stress follows the power law, regardless of the off-axis angle. Hence, it might be possible to estimate the evolution of the viscous strain using the plasticity theory.

The one-parameter plastic potential function proposed by Sun and Chen [11] is adopted to describe the evolution of viscous strain in quasi-UD composites under off-axis tensile loading.In the case of in-plane stresses, it can be written as:

(3) $$2f=\sigma_{22}^{2}+2a_{66}\sigma_{12}^{2}=H\left(\varepsilon_{v}\right)$$

where f is the plastic potential function; $\sigma_{22}$ and $\sigma_{12}$ are the transverse and shear stresses, respectively; $a_{66}$ is an orthotropic coefficient; and H depends on the viscous strain $\varepsilon_{v}$ accumulated and represents the amount of strain hardening experienced by the material system.

The viscous strain increment $d\varepsilon_{x}^{v}$ can be obtained by using the associated flow rule, which is derived as:

(4) $$d\varepsilon_{x}^{v}=d\lambda \frac{\partial f}{\partial \sigma_{ij}}$$

where dλ is a proportionality factor.

The effective stress $\bar{\sigma }$ is defined as:

(5) $$\bar{\sigma }=\sqrt{3f}$$

The associated effective viscous strain increment is given:

(6) $$d{\bar{\varepsilon }}_{v}=\sqrt{\frac{2}{3}{\left(d\varepsilon_{22}^{v}\right)}^{2}+\frac{1}{3a_{66}}{\left(d\gamma_{12}^{v}\right)}^{2}}$$

Under uniaxial and monotonic loading conditions, the effective stress and effective viscous strain increments are related to the applied axial stress and viscous strain as:

(7) $$\bar{\sigma }=\sigma_{x}h\left(\theta \right)$$

(8) $${\bar{\varepsilon }}_{v}=\frac{\varepsilon_{x}^{v}}{h\left(\theta \right)}$$

with

(9) $$h\left(\theta \right)=\sqrt{\frac{3}{2}\left(\sin^{4}\theta +2a_{66}\sin^{2}\theta \cos^{2}\theta \right)}$$

**Figure 10:**
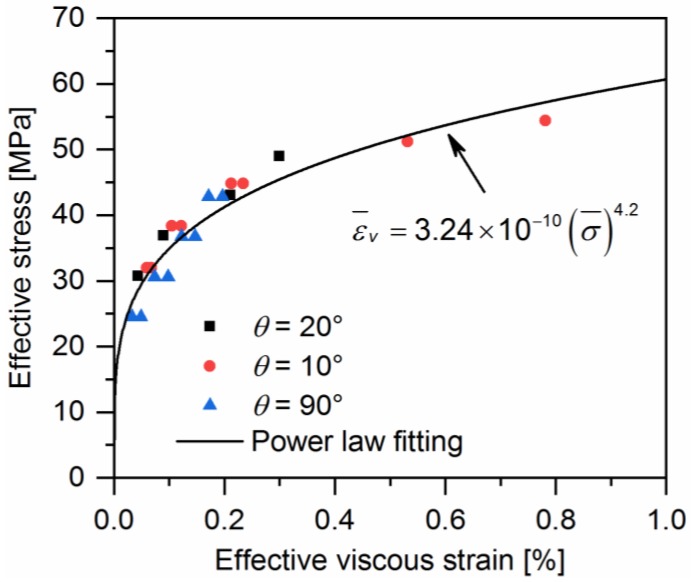
The effective stress-effective viscous strain relationship of quasi-UD E-glass/polypropylene composites.

The effective stress-effective viscous strain relationship of a given material system should be identical, which is independent of the off-axis angle. Apparently, the effective stress-effective viscous strain curve from the 90° off-axis sample is independent of $a_{66}$. Therefore, the effective stress-effective viscous strain curve obtained from the 90° off-axis sample is the master curve. Axial effective stress-effective viscous strain curves that are obtained from different off-axis samples should collapse into a single master curve through adjusting the material parameter $a_{66}$. Figure 10 illustrates the collapsing of curves from different off-axis angles for a single value of $a_{66}$ = 4.5 for quasi-UD E-glass/polypropylene composites. As expected, the single master effective stress-effective viscous strain curve can be fitted with a power law:

(10) $${\bar{\varepsilon }}_{v}=A{\left(\bar{\sigma }\right)}^{n}$$

The material parameters A and n are listed in Table 1.

Based on Equations (7)–(10), the viscous strain of composites under off-axis loading can be described as:

(11) $$\varepsilon_{x}^{v}=A{\left(\sigma_{x}\right)}^{n}{\left(h\left(\theta \right)\right)}^{n+1}$$

### 3.2. Viscoelastic Strain

**Figure 11:**
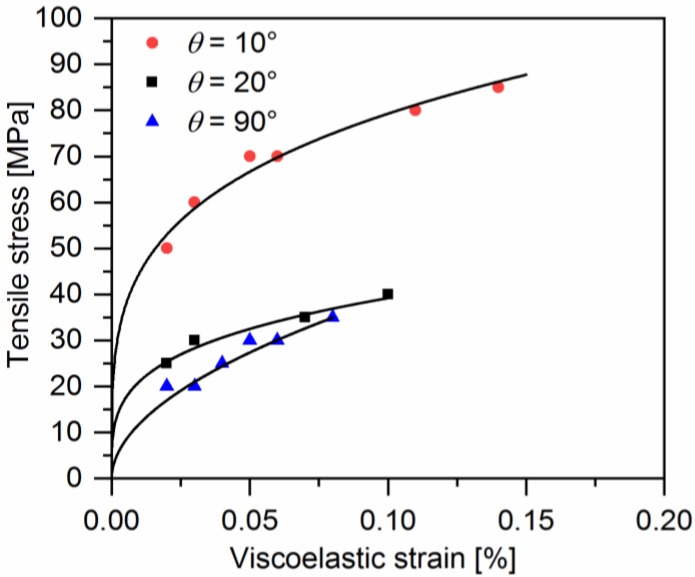
Experimental data of axial stress-viscoelastic strain of a quasi-UD E-glass/polypropylene composite subjected to off-axis tensile loading.

**Figure 12:**
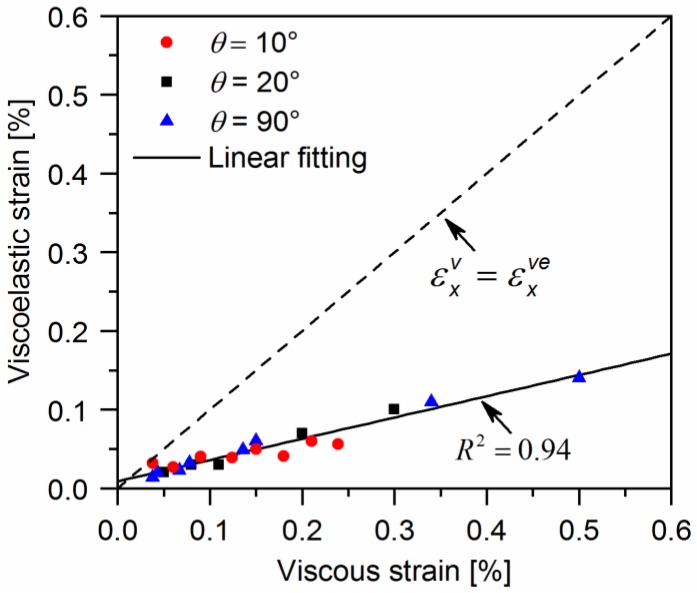
The relationship between the viscous strain and viscoelastic strain of a quasi-UD E-glass/polypropylene composite under off-axis tensile loading.

Figure 11 gives the evolution of viscoelastic strain for off-axis samples as a function of the applied stress. It can be found that the evolution of viscoelastic strain is similar to that of viscous strain. The viscous strain is larger than the viscoelastic strain measured at the same loading stress. The viscoelastic and viscous strains are plotted in Figure 12. It is interesting to find that the relationship between them is almost linear, regardless of the off-axis angle or loading stress. Thus, the viscoelastic strain can be described as:

(12) $$\varepsilon_{x}^{ve}=k\varepsilon_{x}^{v}$$

where k is the ratio of the viscoelastic strain to viscous strain during off-axis tensile loading.

### 3.3. Stiffness Reduction

According to the CDM, the elastic damage variable in Equation (2) is defined as:

(13) $$D=1-\frac{E}{E_{0}}$$

In Equation (13), E is the elastic modulus of damaged material, which can be estimated with the unloading modulus, as shown in Figure 8.Correspondingly, the elastic damage variables of off-axis samples at different loading stresses can be determined.

The elastic damage variables of off-axis samples are described using a Weibull distribution of the effective stress of quasi-UD composites proposed in our previous work [17], which is expressed as:

(14) $$D\left(\bar{\sigma }\right)=1-exp\left(-{\left(\frac{\bar{\sigma }}{\sigma_{e}}\right)}^{n_{e}}\right)$$

The Weibull parameters $\sigma_{e}$ and $n_{e}$ can be determined by the numerical fitting of experimental data.

**Figure 13:**
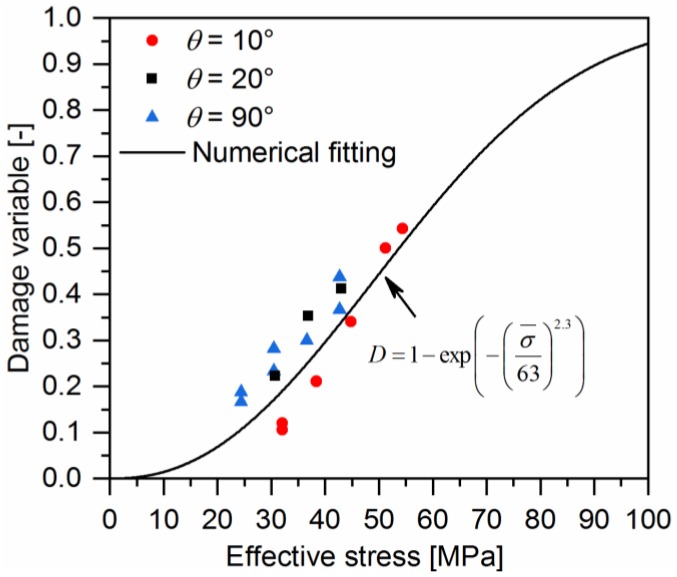
The relationship between the damage variable and the effective stress of a quasi-UD E-glass/polypropylene composite.

The damage variables of off-axis samples and their corresponding effective stresses were obtained by means of Equations (7), (9), and (13). They are listed in Figure 13. As can be seen, the plot of the damage variable-effective stress can be fitted with Equation (14). The fitting parameters are shown in Table 1.

On the basis of the above results, the nonlinear constitutive model for a quasi-UD E-glass/polypropylene composite under off-axis tensile loading can be written as:

(15) $$\varepsilon_{x}=\frac{\sigma_{x}}{E_{0}\exp\left(-{\left(\frac{\sigma_{x}h\left(\theta \right)}{63}\right)}^{2.3}\right)}+A{\left(\sigma_{x}\right)}^{n}{\left(h\left(\theta \right)\right)}^{n+1}+kA{\left(\sigma_{x}\right)}^{n}{\left(h\left(\theta \right)\right)}^{n+1}$$

**Figure 14:**
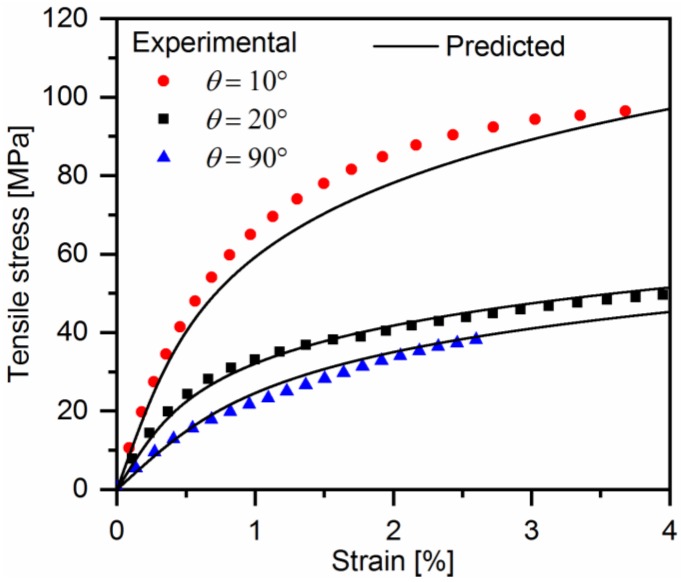
Comparison of predicted axial stress-strain curves and experimental results of a quasi-UD E-glass/polypropylene composite.

Figure 14 illustrates the comparisons between the predicted and observed monotonic stress-strain curves of quasi-UD E-glass/polypropylene composites. The monotonic stress-strain curves are from our previous work [17]. As seen, the predicted monotonic nonlinear responses show a good agreement with the experimental results.

## 4. Conclusions

To identify the individual contribution of viscoelastic and viscous deformation to the global nonlinear response, multilevel cyclic loading-unloading recovery tests were performed on quasi-UD E-glass/polypropylene composites. The main conclusions of this present work are:

1. The overall strains of quasi-UD E-glass/polypropylene composites under off-axis tensile loading come from elastic, viscoelastic, and viscous strains.
2. The evolutions of both viscoelastic and viscous strain are dependent on loading stress and the off-axis angle, which may be attributed to different deformation and fracture mechanisms in off-axis tension.
3. The viscous strain shows a linear relationship with the evolution of viscoelastic strain, regardless of the loading stress or fiber orientation.
4. Based on experimental results, a new material model is developed to describe the nonlinear stress-strain curves of quasi-UD composites under off-axis tensile loading, which account for viscoelastic, viscous deformation, and stiffness degradation. In the proposed model, the plastic deformation is decomposed into two parts, i.e., time-dependent reversible plastic strain (viscoelastic strain) and irreversible strain (viscous strain). The plasticity theory is employed to describe the evolution of the viscous strain with loading stress. The viscoelastic strain is represented as a linear function of viscous strain. In addition, the evolution of time-independent reversible strain (elastic strain) can be estimated with the Weibull distribution of the effective stress of composites. The predictions agree well with the experimental results.

## Figures and Tables

**Table 1:** Parameters in the model (Equation (15)).

*a*_66_	*A* (MPa)*^−n^*	*n*	*k*	*σ_e_*(MPa)	*n_e_*
4.5	3.24 × 10^−10^	4.2	0.27	63	2.3
